# Significant Decline in Galactomannan Signal during Storage of Clinical Serum Samples

**DOI:** 10.3390/ijms140712970

**Published:** 2013-06-24

**Authors:** Gemma L. Johnson, Shah-Jalal Sarker, Kate Hill, Dimitris A. Tsitsikas, Amelie Morin, Stephen A. Bustin, Samir G. Agrawal

**Affiliations:** 1 Blizard Institute of Cell and Molecular Science, Queen Mary University of London, London E1 2AT, UK; E-Mail: gemma.johnson@bartshealth.nhs.uk; 2 Centre for Experimental Cancer Medicine, Barts Cancer Institute, Queen Mary University of London, London EC1M 6BQ, UK; E-Mail: s.sarker@qmul.ac.uk; 3 Division of Infection, Barts Health NHS Trust, London E1 2ES, UK; E-Mail: kate.hill@bartshealth.nhs.uk; 4 Department of Haemato-Oncology, St Bartholomew’s Hospital, London EC1A 7BE, UK; E-Mails: dimitris.tsitsikas@bartshealth.nhs.uk (D.A.T.); amelie.morin@bartshealth.nhs.uk (A.M.); 5 Postgraduate Medical Institute, Faculty of Health, Social Care & Education, Anglia Ruskin University, Chelmsford CM1 1SQ, UK; E-Mail: stephen.bustin@anglia.ac.uk

**Keywords:** invasive aspergillosis, platelia, galactomannan

## Abstract

Galactomannan (GM) is widely used for detection of invasive aspergillosis in high-risk haemato-oncology patients. Recent publications have reported a lack of repeatability of GM detection. The objective of this retrospective study was to assess the repeatability of GM levels during storage of clinical samples. In a GM screening strategy, positive sera were repeat tested as per manufacturer’s recommendations. Short-term (ST) storage of samples was at +4 °C while long-term (LT) storage was at −80 °C. Bronchoalveolar (BAL) fluid was also repeating tested after ST storage and LT storage. Wilcoxon Signed Ranks Test was employed to assess the repeatability of GM levels. In a subset of 14 GM positive sera, repeat testing was performed on both the original serum and ethylenediaminetetraacetic acid (EDTA) pre-treated sample. There was a significant reduction in GM signals on repeat testing following ST storage (median GM index: 0.65 *vs.* 0.19; *p* < 0.001) and LT storage (median GM index: 0.56 *vs*. 0.10; *p* < 0.001) of serum samples. Of samples that were initially GM positive, an average GM index reduction of 50% was seen, with approximately two-thirds becoming GM negative on repeat testing of the same sample. In contrast, GM signal loss was not seen on repeat testing of BAL fluid following ST or LT storage. When GM positive serum samples were repeat tested using EDTA pre-treated serum from the first step of the testing protocol, all samples remained GM positive. In contrast, when the same samples were repeat tested from the original collected serum, 9 samples (64%) became GM negative. The significant reduction in GM signals during ST and LT storage of serum samples has implications for clinical management. Although the reasons for GM decline are unknown, they occur prior to the EDTA pre-treatment stage, indicating that the time from phlebotomy to testing should be minimized. BAL fluid GM index values remain stable.

## 1. Introduction

Invasive pulmonary aspergillosis (IA) remains a life-threatening opportunistic invasive mycosis in immunocompromised patients [1]. The Platelia Aspergillus enzyme immunoassay (PA-EIA, Bio Rad, Marnes-la-Coquette, France) provides non-invasive, quantitative Aspergillus-specific detection and is widely used to detect serum galactomannan (GM) [2]. There is little information on the repeatability of GM Index results on repeat testing of the same sample after a period of sample storage. One study retrospectively evaluated GM concentrations in stored serum after a period of four years, reporting a lower reactivity in 20% of samples on retesting [3]. However, analysis of “real-time” results has also demonstrated significant between-run variability when testing is repeated on the same sample [4], underlying the importance of corroborating a positive result by retesting of the same sample. Two recently published articles have reported a lack of intra-laboratory repeatability with the PA-EIA test for GM detection. Oren *et al.* report a lack of repeatability when GM positive samples are retested within six days [5], with lower values in 29/34 (85%) repeats. Bizzini *et al.* [6] report a similar reduction in reactivity of samples on retesting of positive samples within three days, with lower values in 23/29 (79%) repeats. These observations raise doubts about the interpretation of data from frozen/thawed samples, as well as samples tested in “real-time” for patient management. The aim of this retrospective study was to clarify the effects of sample storage on the repeatability of GM levels using the PA-EIA.

## 2. Results and Discussion

### 2.1. Sample Results (Scatter Plot)

**Figure 1:**
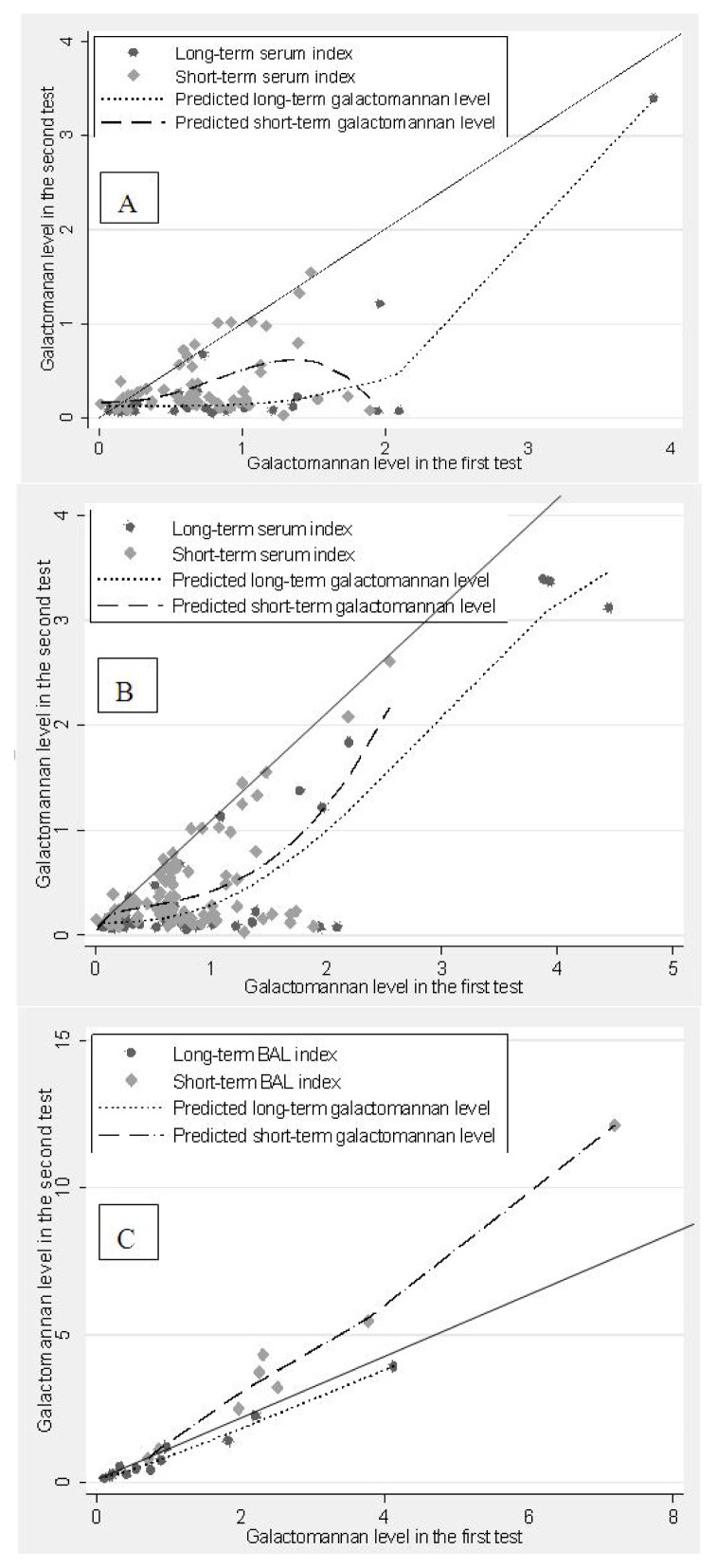
Scatter plot with fractional polynomial lines for reduction in Galactomannan (GM) level. Diagonal solid lines indicate the line of equality. (**A**) GM serum level excluding repeat sera from patients with multiple samples; (**B**) GM serum level including multiple samples from the same patient; (**C**) GM Bronchoalveolar (BAL) level (no repetition) during short-term and long-term storage.

Figure 1 shows that there was a clear loss of GM signal in both the short-term (ST) and long-term (LT) serum, with the signal loss more apparent in the plot including only the first sample taken from each patient (Figure 1A) than in the plot including all serum data (Figure 1B). Repeated samples from the same patient may mask the loss of signal in both ST and LT stored sera. Loss of GM signal was higher in LT than ST stored samples. BAL fluid samples did not show GM signal loss following either in ST or LT storage (Figure 1C). Note that the removal of the two outlier values from Figure 1A,C does not change the shape of the relationships significantly.

#### 2.1.1. Hypothesis Testing

Serum samples (without repetition): The Wilcoxon Signed Rank Sum Test applied to the first sample from each patient, showed statistically significant reductions in GM signals both in the ST storage (*n* = 65, median GM1 index: 0.65, median GM2 index: 0.19; *p* < 0.001) and in the LT storage (*n* = 35, median GM1 index: 0.56, median GM2 index: 0.10; *p* < 0.001), as shown in Table 1. In total, 44/65 (68%) ST and 31/35 (89%) LT serum samples showed a reduction in GM signal upon repeat testing.

In the ST serum group, 65.2% of samples that were initially GM positive became GM negative on repeat testing; with an average GM signal reduction of 50.2% in samples that were initially GM positive. In the LT serum storage group, 67.9% of GM positive samples became GM negative on repeat testing; with an average GM signal reduction of 49.3%.

To ascertain the stage at which GM signal loss occurred, repeat testing in a subset of GM positive samples (*n* = 14) was performed following ST storage from the original serum sample or using the EDTA pre-treated sample. All EDTA pre-treated samples remained positive. In contrast 9/14 (64%) of repeats from the original serum sample became negative.

#### 2.1.2. Assay Repeatability

**Figure 2:**
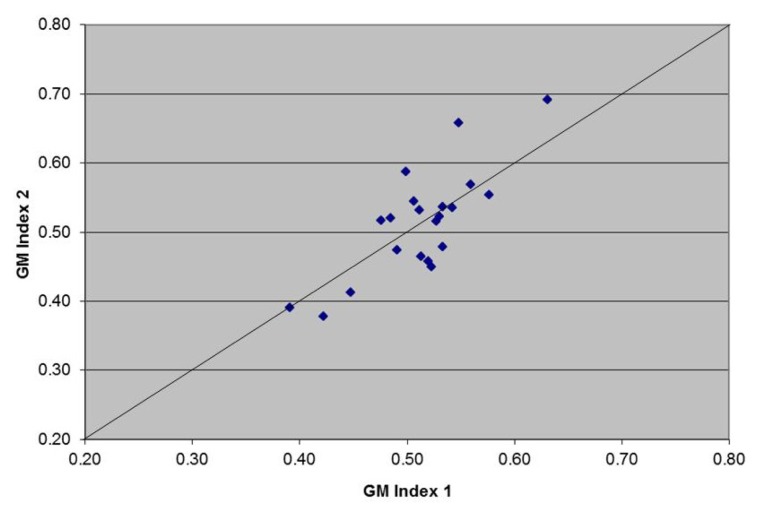
Scatter plot for repeatability of the threshold control: Controls provided with the Platelia Aspergillus enzyme immunoassay (PA-EIA) kit were loaded in duplicate wells (GM Index 1 and GM Index 2) over 40 randomly selected assay runs. Diagonal solid line indicates the line of equality.

The repeatability of the OD of the threshold control run in duplicate over 40 randomly selected assays is shown in Figure 2. The duplicate values of the OD were repeatable as most of them are close to the line of equality and all measurements were within the recommended limits (OD 0.3–0.8). The mean OD of the 40 threshold control measurements was 0.49, with range 0.33–0.69. The coefficient of variation for duplicate measures was 7.09, which confirms repeatability.

### 2.2. Discussion

The international EORTC/MSG (European Organization for Research and Treatment of Cancer/Invasive Fungal Infections Cooperation Group and the National Institute of Allergy and Infectious Diseases Mycoses Study Group) mycological criteria for diagnosing IA [7] include serum and BAL fluid GM results as key diagnostic criteria. These are routinely obtained using the PA-EIA, which provides non-invasive, quantitative Aspergillus-specific detection for serum GM. Recent reports have raised concerns with regard to the repeatability of GM levels. This report confirms these doubts and broadens these findings by establishing a lack of repeatability of serum GM testing of the same sample. A clinically significant reduction in GM reactivity was recorded, with two thirds of GM positive sera becoming negative following ST or LT storage. This phenomenon was not seen with repeat GM testing of BAL fluid following ST or LT storage. We have published our rate of invasive fungal disease (IFD) using the EORTC/MSG criteria of probable/proven IFD, which are usually regarded as representing true cases, as only 8/589 episodes (1.4%) by 2008 and 20/589 (3.5%) by 2002 criteria [8]. It has been previously reported by Furfaro *et al.* [9] that the decrease in GM values after retesting was much more pronounced in those samples with initially false positive GM results. Clearly, in our practice, the phenomenon of GM signal loss cannot be correlated to the EORTC/MSG criteria of IFD given the much higher frequency GM signal loss in both ST and LT data sets.

The OD value of the threshold control is critical in the calculation of the GM index value of clinical samples. Repeatability of the OD of the threshold control was confirmed by the CV for duplicate measures. These data indicate that technical issues per se cannot explain the observed GM signal loss in clinical samples. Furthermore, extensive review of the technical performance of the assay by different operators was undertaken, in conjunction with Bio-Rad, including monitoring of heating block temperature and plate washer performance. No concerns were identified. Although environmental contamination is a risk during testing, our data are not consistent with contamination, which would lead to random increases or decreases with repeat testing. In contrast, the presented data show a consistent pattern of signal loss, with the first reading being higher than the repeat. In addition, sporadic repeat testing of GM negative serum samples never gave a positive result.

The repeatability of the OD of the threshold control suggests that the observed GM signal loss was specific to clinical serum samples. Interestingly, following ST storage of sera, the GM signal loss was not seen if the repeat was performed using EDTA pre-treated samples from the first step of the testing protocol, all of which remained positive. Although the mechanisms of GM decline in clinical serum samples are unclear, they appear to occur in the original serum sample prior to the EDTA pre-treatment stage. The data does not allow us to specify an acceptable storage time without major signal loss, but we can conclude that serum signal loss is associated with storage, and that it is therefore important to minimise the time from phlebotomy to the EDTA stage of processing. Further work needs to be done to determine whether short term storage without major signal loss is possible. The focus of our further investigations is on the stability of *in vivo* serum GM *versus* purified control GM, the impact of potential enzymatic epitope degradation/masking, as well as the correlation with any prescribed anti-microbial agents and other intravenous drugs.

## 3. Experimental Section

### 3.1. Clinical Samples

Serum and broncho-alveolar lavage (BAL) fluid samples were collected from patients in the Division of Haemato-Oncology at St Bartholomew’s Hospital undergoing serum GM monitoring in a screening strategy throughout their episode of intensive chemotherapy. Samples were sent, and GM detection was performed by PA-EIA, twice weekly. Techniques were carried out as recommended by the manufacturer. Results were expressed as a GM index value—The ratio of the sample optical density (OD) divided by the mean OD of the 2 threshold controls. Serum samples were scored as positive if the GM index was >0.5. In our institution, it is routine practice to confirm a positive result (GM1) by repeat testing (GM2) from the original sample, as recommended by the manufacturer. For GM negative serum samples, repeat testing was sporadically performed for performance analysis.

Short-term (ST) storage at +4 °C: Microbiology laboratory records from February 2009 to March 2010 were filtered to exclude GM data (i) for non-serum samples and (ii) samples repeat-tested after greater than 48hours. A total of 109 serum samples (from 65 different patients) and 8 BAL fluid samples were identified.

Long-term (LT) storage at −80 °C: A total of 54 serum samples (from 35 different patients) and 12 BAL fluid samples were collected from July 2005 to September 2007, GM tested and sample aliquots were stored at −80 °C. These samples were repeat tested after 2–4 years storage at −80 °C in 2009. In total, 183 clinical samples were included in this study.

### 3.2. Analysis of Clinical Data

Data from serum testing are presented on a scatter plot, with fractional polynomial regression lines for each storage type. As the relationship between the two test results was found not to be linear, fractional polynomial regression was the best choice to capture the exact relationship between them. Due to routine twice-weekly screening of high-risk patients, more than one sample was collected for most of the patients. As first and second test values are likely to be correlated, the Wilcoxon Signed Rank Sum Test was used to test the GM signal loss both in the ST as well as LT serum samples based on the first sample taken from each patient. Probability values <0.05 were defined as significant based on a 2-tailed test. All calculations were carried out using the statistical software package Intercooled STATA 10.1 (Stat Corp, College Station, TX, USA, 2008).

### 3.3. Assay Repeatability

In order to determine the intra- and inter-assay variability, the OD value of the threshold control, loaded in duplicate, was recorded for 40 randomly selected assay runs. The mean OD was calculated, together with range. Inter-assay repeatability of the threshold control was calculated by using the Jones and Payne [10] coefficient of variation (CV) for duplicate measures.

## 4. Conclusions

These observations suggest that: (i) caution should be exercised when testing serum samples after a period of storage, including samples stored at −80 °C; (ii) the interpretation of GM negative results obtained after delays in processing may be problematic; (iii) careful coordination is required between the laboratory and clinical teams to minimise the time from sampling to testing; (iv) a better understanding of GM kinetics is necessary to address the impact of sample processing and storage times on detectable GM concentrations. Furthermore, given a wide range of sensitivity and specificity data plus multiple causes of false-positive results for the PA-EIA reported in the literature, a prudent approach to clinical utilization of GM testing would be to combine the PA-EIA with other markers of *Aspergillus* in a diagnostic algorithm [11,12].

## Figures and Tables

**Table 1:** Results of the Wilcoxon Signed Rank Sum Test.

	Sample size	Samples with GM reduction	Median GM1 index	Median GM2 index	*p*-value
Short-Term Serum	65	44	0.65	0.19	<0.001
Long-Term Serum	35	31	0.56	0.10	<0.001
